# Epidemiology of Classical Hodgkin Lymphoma and Its Association with Epstein Barr Virus in Northern China

**DOI:** 10.1371/journal.pone.0021152

**Published:** 2011-06-10

**Authors:** Xin Huang, Ilja Nolte, Zifen Gao, Hans Vos, Bouke Hepkema, Sibrand Poppema, Anke van den Berg, Arjan Diepstra

**Affiliations:** 1 Department of Pathology and Medical Biology, University Medical Center Groningen, University of Groningen, Groningen, The Netherlands; 2 Department of Pathology, Health Science Center, Peking University, Beijing, China; 3 Unit of Genetic Epidemiology and Bioinformatics, Department of Epidemiology, University Medical Center Groningen, University of Groningen, Groningen, The Netherlands; 4 Department of Laboratory Medicine, University Medical Center Groningen, University of Groningen, Groningen, The Netherlands; University of Nebraska – Lincoln, United States of America

## Abstract

**Background:**

The incidence of classical Hodgkin lymphoma (cHL) and its association with Epstein-Barr virus (EBV) varies significantly with age, sex, ethnicity and geographic location. This is the first report on epidemiological features of cHL patients from Northern regions of China. These features are compared to data from a previously published Dutch cHL population.

**Methodology/Principal Findings:**

157 cHL patients diagnosed between 1997 and 2008 in the North of China were included after histopathological re-evaluation. The Dutch population-based cohort consisted of 515 cHL patients diagnosed between 1987 and 2000. EBV status was determined by in situ hybridization of EBV- encoded small RNAs. In the Chinese population, tumor cells of 39% of the cHL patients were EBV+ and this was significantly associated with male sex, mixed cellularity subtype and young age (<20 y). The median age of the Chinese patients was 9 years younger than that of the Dutch patients (28 y vs. 37 y). In addition, the age distribution between the two populations was strikingly different in both the EBV+ subgroups (p<0.001) and the EBV- subgroups (p = 0.01). The mixed cellularity subtype was almost 3x more frequent amongst the Chinese (p<0.001).

**Conclusion/Significance:**

CHL patients from Northern regions of China show a distinctive age distribution pattern with a striking incidence peak of EBV+ mixed cellularity cases among children and adolescents and another high incidence peak of EBV- nodular sclerosis cases in young adults. In comparison to Dutch cHL patients there are pronounced differences in age distribution, subtype and EBV status, presumably caused by complex gene-environmental interactions.

## Introduction

Classical Hodgkin lymphoma (cHL) is a heterogeneous malignancy with a complex etiology and epidemiology. In general, cHL accounts for about 1% of all cancers and ∼30% of the lymphoid malignancies worldwide [1]. Epidemiologic studies of cHL demonstrate a remarkable diversity of the incidence according to age, sex, ethnic background, geographic location and socioeconomic status [2], [3]. The highest incidence was reported among Caucasians, followed by African Americans and Hispanics, and the lowest incidence was found in Orientals [3]. Data from the International Agency for Research on Cancer (http://globocan.iarc.fr/) shows a nearly 6-fold difference between Western Europe and East Asia with an incidence of 2.3 and 0.4 per 100,000 inhabitants per year respectively in 2008. A genetic explanation for this difference has been shown in a multi-ethnic study of cHL in the United States that reported the lowest incidence rate in Asian immigrants in comparison to other ethnic origins [4]. However, a trend of increasing incidence of cHL was reported among Chinese immigrants in western countries [4], [5], suggesting an influence of westernization. The incidence pattern by age was also shown to be different between Caucasian and Oriental populations [6], [7]. Western populations typically have a bimodal age distribution with two peaks near 25 and 60 ys. In Orientals, a first incidence peak usually presents in childhood with a second peak in the elderly, although in Japanese cHL patients the early incidence peak was reported to be absent [4], [8].

Epstein-Barr virus (EBV) is present in the tumor cells in a proportion of patients and EBV is accepted as a causal agent in these patients [7], [9]. There is a striking variability in the percentage of EBV involved cases between racial groups and geographic locations [2], [10]. The proportion of EBV involvement is almost 100% in Hispanic cHL patients [11], much lower in Caucasians (20∼40%) [2], [12] and intermediate in Orientals [13], [14]. In general, the EBV association with cHL is related to age, being the strongest in children and the elderly [15], [16]. In addition, male sex and the mixed cellularity (MC) histological subtype are associated with EBV+ cHL worldwide [15], [17].

The current study was undertaken to investigate the epidemiological characteristics of cHL patients from Northern China. In addition, these characteristics were compared to data from a previously reported Dutch cHL patient population [18].

## Materials and Methods

### Patient selection and data collection

157 cHL patients were included in this study [19]. These patients resided in the Northern area of China and were diagnosed with cHL during the period of 1997 to 2008 at the Dept. of Pathology, Health Science Center, Peking University (n = 78), Zhanye Regional Hospital, Gansu Province (n = 22), First Hospital of Jilin University (n = 14), Beijing Air Army General Hospital (n = 14), Shougang Hospital, Peking University (n = 13) and a number of other smaller hospitals (1 to 3 patients per hospital; n = 16). For all patients we retrieved the original data from the pathology database, including histological subtype, patients' sex and age at the time of diagnosis. Ethical approval for this study was not required by these institutions as the experiments carried out did not relate to patients privacy or treatment. Research was conducted adhering to the Declaration of Helsinki and according to Dutch regulations (http://www.federa.org/). The institutional review board (the Medical Ethics Review Board of the University Medical Center Groningen) specifically waived the needs for ethics approval and consent.

### Histopathological re-evaluation

Formalin fixed paraffin embedded tissue blocks from 157 patients were available and were used to cut slides for haematoxylin and eosin staining and for determining EBV status. Histological re-evaluation was done according to the most recent WHO classification system [1] which categorizes cHL into four histological subgroups, i.e. nodular sclerosis (NS), mixed cellularity (MC), lymphocyte rich (LR) and lymphocyte depletion (LD). Cases without enough tissue for proper evaluation of the background architecture were designated as cHL not otherwise specified (NOS).

### EBV status

Presence of EBV in tumor cells was determined by EBER in situ hybridization (ISH) with a fluorescein-conjugated PNA probe specific for the EBV-encoded EBER RNAs (DAKO, Glostrup, Denmark) using standard laboratory protocols. Appropriate positive and negative controls were included in all analyses.

### Statistical analysis

Differences between EBV+ and EBV- groups in relation to age, sex and histopathological subtype were assessed by Chi square test, Fisher's exact test or Mann Whitney U test. The data were analyzed with SPSS for Windows, version 17.0. A p-value <0.05 was considered significant.

## Results

### Histopathological re-evaluation and EBV status

The 157 cHL cases were re-evaluated for histological subtype. The subtype could not be unequivocally determined in 29 (18%) cases and was designated cHL-NOS. Six and 9 of these cases were previously classified as LR and MC subtype respectively. Discrepancies in the classification were observed in 5 out of 13 LR cases and 6 out of 57 MC cases which were reclassified as MC and NS subtype respectively. All original NS diagnoses were re-evaluated as NS subtype. A consistent classification was achieved for 117 of 128 (91.4%) cHL cases.

NS was the most common subtype in the Chinese patient population, accounting for 61% of cases (n = 78), followed by MC with 36% (n = 46) and LR with 3% (n = 4) of cases. The lymphocyte depleted subtype was not represented. EBER ISH was positive in 39% of Chinese cHL patients (n = 62; Table 1).

**Table 1:** Distribution of age, sex and histology by EBV status in Chinese cHL patients.

	all patients		EBV+		EBV-		EBV+ vs. EBV-
	n = 157		n = 62		n = 95		p-value
	**n**	**%**	**n**	**%**	**n**	**%**	
**sex**							
male	101	64	47	76	54	57	0.015*
female	56	36	15	24	41	43	
**histological subtype**							
NS	78	61	12	25	66	82	<0.001^†^
MC	46	36	34	71	12	15	
LR	4	3	2	4	2	3	
NOS	29		14		15		
**median age (range)**	28 (4–74)		31 (4–74)		28 (8–74)		n.s.^‡^

*Chi square test, ^†^ Fisher's exact test, NOS cases were excluded, ^‡^ Mann Whitney U test, NS indicates nodular sclerosis; MC, mixed cellularity; LR, lymphocyte rich; LD lymphocyte depletion; NOS, not otherwise specified; n.s., not significant.

### Distribution of age, sex and histological subtypes

The male to female ratio was 1.8 in the total group, 3.1 in the EBV+ group and 1.3 in the EBV- group (p = 0.015). The MC subtype was more common in EBV+ cHL patients (71%) and the NS subtype was more common in the EBV- group (82%) (p<0.001). Although the median age of cHL patients in the EBV+ group did not differ much from that of the EBV- cHL patient group (31 years, range: 4–74 ys and 27 years, range: 8–74 ys respectively) (Table 1), the 10 years age group distribution between EBV+ and EBV- patients did show a highly significant difference (p<0.001). Patients in the age groups from 0 to 10 were significantly more often EBV+ (p = 0.003), while patients aged 21 to 30 were significantly more often EBV- (p<0.001).

### Comparison between Chinese and Dutch cHL patients

Characteristics of the Dutch cHL population were consistent with the epidemiology of cHL in Western Europe (Table 2). There was a small difference in the sex distribution between Dutch and Chinese cHL patients with a male to female ratio of 1.8 in the Chinese and 1.4 in the Dutch population. In the EBV+ group males were overrepresented in the Chinese as compared to the Dutch population (male to female ratio of 3.1 vs. 1.8); however, these differences were not significant. In the EBV- group the Chinese male to female ratio was comparable to the Dutch (1.3 vs. 1.2).

**Table 2:** Distribution of age, sex and histology by EBV status in Dutch cHL patients.

	all patients		EBV+		EBV-		EBV+ vs. EBV-
	n = 515		n = 181		n = 334		p-value
	**n**	**%**	**n**	**%**	**n**	**%**	
**sex**							
male	298	58	118	65	180	54	0.013*
female	217	42	63	35	154	46	
**histological subtype**							
NS	413	83	114	66	299	92	<0.001^†^
MC	63	13	48	28	15	4	
LR	12	2	3	2	9	3	
LD	9	2	7	4	2	1	
NOS	18		9		9		
**median age (range)**	37 (8–94)		43 (8–94)		34 (9–90)		<0.001^‡^

*Chi square test, ^†^ Fisher's exact test, NOS cases were excluded, ^‡^ Mann Whitney U test, NS indicates nodular sclerosis; MC, mixed cellularity; LR, lymphocyte rich; LD lymphocyte depletion; NOS, not otherwise specified.

In the Chinese patients, the MC subtype was much more frequent than in the Dutch patients (36% vs. 13%) (p<0.001) (Table 3). In both populations the MC subtype was highly and equally associated with EBV positivity (Chinese 74% vs. Dutch 76%). The NS subtype was less often EBV associated in the Chinese as compared to the Dutch cHL patients (15% vs. 28%) (p = 0.024).

**Table 3:** Differences in subtype distribution in Chinese and Dutch cHL patients.

	Chinese		Dutch		
	n	%	n	%	p-value
**all patients**	157		515		.
NS	78	61	413	83	<0.001*
MC	46	36	63	13	
LR	4	3	12	2	
LD	0	0	9	2	
NOS	29		18		
**all EBV+ patients**	62		181		
NS	12	25	114	66	<0.001*
MC	34	71	48	28	
LR	2	4	3	2	
LD	0	0	7	4	
NOS	14		9		
**young patients (4–15 ys)**					
NS	5	25	21	95	<0.001^†^
MC	14	70	1	5	
LR	1	5	0	0	
NOS	4		1		
**young EBV+ (4–15 ys)**					
NS	3	18	8	89	<0.001^†^
MC	14	82	1	11	
LR	0	0	0	0	
NOS	4		1		

*Chi square and ^†^ Fisher's exact test for NS and MC; NS indicates nodular sclerosis; MC, mixed cellularity; LR, lymphocyte rich; LD lymphocyte depletion; NOS, not otherwise specified.

**Figure 1:**
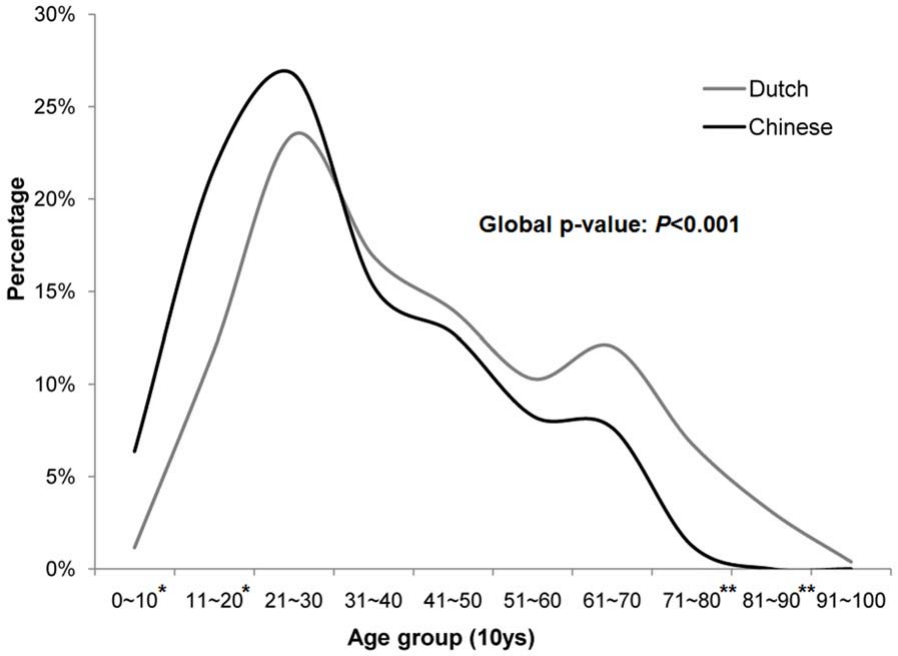
Age distribution of Dutch and Chinese cHL patients in consecutive 10-year intervals. Age distribution is shown for 157 patients from Northern regions of China (black) and 515 patients from the Northern part of the Netherlands (grey). The two curves exhibit a somewhat similar trend with significant differences at the first, second, eighth and ninth decade (* = p<0.01, ** = p<0.001) between the Chinese and the Dutch population (6% vs. 1%, 22% vs. 12%, 1% vs. 7% and 0% vs. 3% respectively). The Chinese population has more patients among the first three decades as compared to the Dutch population, both having the highest peak in the third decade. The Chinese population does not show a second incidence peak at around 60 years.

**Figure 2:**
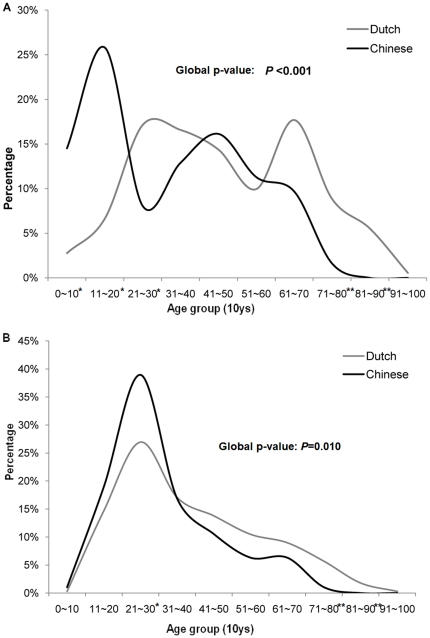
Age distribution of Dutch and Chinese cHL patients stratified by EBV status. A represents the comparison of the age distribution in the two EBV+ cHL subpopulations. Chinese EBV+ cHL patients (black) exhibited a significant single peak in the first and second decade with a maximum percentage of 26% (* = p<0.01). In contrast, Dutch EBV+ cHL patients (grey) demonstrated a clear bimodal age distribution pattern with two peaks occurring in the third and seventh decade respectively with a similar incidence for both peaks (17% and 18%). B shows the age distribution curve of the EBV- cHL patients. There is a single incidence peak in the third decade for both populations, which is more pronounced in the Chinese population (39% vs. 27%).

The median age of Chinese patients (28 ys, range: 4–74 ys) was 9 years younger than that of the Dutch population (37 ys, range: 8–94 ys) (p<0.001) and the age distribution in 10-year intervals was significantly different (p<0.001) (Figure 1, Table 4). Similar to the Chinese patients, stratification by EBV status showed significant differences in median age between EBV+ (43 ys, range: 8–94) and EBV- (33 ys, range: 9–90) Dutch cHL patients (p<0.001). Comparison of the EBV+ Chinese patient group with the EBV+ Dutch patient group showed a striking difference in the age distribution (p<0.001) (Figure 2A, Table 4). In young Chinese patients (younger than 16 years), there was a high number of MC subtype (14 out of 20) and all of these MC cases were EBV associated (Table 3). In addition, the age distribution of the EBV- Chinese patients was significantly different from the EBV- Dutch patients (p = 0.01) (Figure 2B, Table 4).

**Table 4:** Differences in age distribution in Chinese and Dutch cHL patients.

		Chinese			Dutch		
	n	age		n	age		p-value*
		median	range		median	range	
**all patients**	157	28	4–74	515	37	8–94	<0.001
**EBV−**	95	28	8–74	334	34	9–90	0.01
**EBV+**	62	31	4–74	181	43	8–94	<0.001

*Mann Whitney U test.

## Discussion

Epidemiological cHL studies have shown significant variations in age and EBV positivity in relation to ethnic background and geographic location. Asians are known to have the lowest incidence of cHL and an intermediate level of EBV association [3]. Previously, a few studies reported on cHL epidemiology in Taiwan and Hong Kong, populations that are closely related to the Southern Chinese population [13], [20]–[22]. Importantly, the Northern Chinese population is genetically different from the Southern Chinese population, especially in the distribution of Human Leukocyte Antigen (HLA) types. Undifferentiated nasopharyngeal carcinoma (UNPC), another EBV-associated malignancy with a latent EBV expression pattern similar to EBV+ cHL, is endemic in Southern China but not in Northern China. Previous studies have proposed that geographic differences in the allele frequency of the UNPC susceptibility allele HLA-A*02∶07 might well explain the high incidence of UNPC in the Southern Chinese population. Thus, differences in cHL incidence and EBV association might also be expected in different Chinese regions. The main aim of this study was to analyze the epidemiology of cHL in patients from the Northern part of China, a population that has not been studied before.

Previous studies found a relatively high level of EBV association in cHL patients from Hong Kong (65%, n = 23, EBER ISH), China (61%, n = 28, EBER ISH) and Taiwan (63%, n = 70, EBER ISH and LMP1 immunohistochemistry combined) [13], [14], [21]. These frequencies are higher than the 39% of EBV positivity identified in the present patient cohort (n = 157). Besides the population size, differences in EBV prevalence might also be influenced by intra-ethnic environmental and genetic variations. Consistent with previous findings in Chinese and other ethnic groups of cHL patients, the incidence of EBV+ cHL was significantly higher in males and the MC subtype [2].

A shift in the histological classification upon histopathological re-examination was observed in 8.6% (n = 11) of the cases, partly explained by changes in the WHO classification guidelines. Agreement was specifically poor for the LR and MC subtypes. Due to the lack of enough tissue for evaluating the background architecture, 15 cases previously diagnosed as LR or MC subtype were re-assigned as cHL-NOS. Overall, the histological distribution was similar to those reported in previous Chinese populations from Hong Kong and Taiwan [13], [23].

Until now, there are four studies that investigated the age distribution of cHL in the Chinese population [13], [20]–[22]. Three studies, including two from Hong Kong (n = 23 and n = 92) [13], [20] and another one from Taiwan (n = 70) [21], identified a bimodal age distribution with two peaks at the second and sixth decades respectively, whereas another study from Taiwan (n = 42) [22] found a single age peak at the 3rd decade. Consistent with this last study, we also found a unimodal age distribution with a distinctive peak incidence occurring at the 3rd decade. Only two of the four studies (Hong Kong and Taiwan) included EBV status into the analyses [13], [21]. Our results were consistent with these studies for EBV- cHL patients with a single incidence peak in young-adults. However, our EBV+ cHL patients did not show an incidence peak in the elderly, in contrast to both other studies.

Comparison of the Chinese and Dutch cHL patient groups revealed a significant inter-ethnic difference in the age distribution in the total populations and also in the EBV+ and EBV- subgroups separately. A bimodal pattern for the age distribution of cHL in general as well as in the EBV+ subgroup is considered typical for the Western population and this was also present in our Dutch cohort. It has been reported that in the Asian cHL population, an early peak should be less obvious and at a younger age compared to the Western population, whereas a second peak at older age should be similar [4], [8]. Intriguingly, our Chinese population had an evident early peak similar to the Dutch population but lacked the peak at older age. The relative lack of elderly patients in our study might partially be explained by a lower life expectancy in China as compared to the Netherlands. In addition, there might be a bias of elderly patients being diagnosed in smaller, rural hospitals that were not included in this study. It is unclear whether these potential effects could have affected an incidence peak in the 61 to 70 years age group.

According to the literature, EBV+ Asian cHL shows a similar age distribution as EBV- Asian cHL. However, in the present study EBV+ cHL had a different and pronounced incidence peak at adolescence and was strongly associated with the MC subtype. This incidence peak of EBV+ cHL at young age closely resembles that seen in various developing regions in the world, including South America, Mexico and the Middle East and is associated with a low socioeconomic status and early exposure to EBV [24]–[30]. In both the Chinese and the Dutch populations a clear peak for EBV- NS patients was observed at the 3rd decade. This peak was higher in the Chinese population than in the Dutch population (39% vs. 27%), which is surprising because this peak has been associated with developed, industrialized populations [31]. Thus, the epidemiology of our cHL patient cohort from the Northern part of China shows characteristics of both a developing and a well-developed population.

In conclusion, we demonstrate a distinctive age distribution pattern in a large group of Northern Chinese cHL patients with a striking incidence peak among young adults. Moreover, we identified large inter-ethnic differences in the distribution of age, EBV status and histological subtype in comparison to a Caucasian population. Both genetic and environmental factors are expected to play a role in these differences.
